# Translation, cross-cultural adaptation, and validation of the Italian language Forgotten Joint Score-12 (FJS-12) as an outcome measure for total knee arthroplasty in an Italian population

**DOI:** 10.1186/s12891-019-2985-2

**Published:** 2020-01-11

**Authors:** Valerio Sansone, Peter Fennema, Rachel C. Applefield, Stefano Marchina, Raffaella Ronco, Walter Pascale, Valerio Pascale

**Affiliations:** 1 Department of Orthopaedics, Università degli Studi di Milano, Via Festa del Perdono, 7, 20122 Milan, MI Italy; 2 IRCCS Istituto Ortopedico Galeazzi, Via Riccardo Galeazzi, 4, 20161 Milan, MI Italy; 3 AMR Advanced Medical Research GmbH, Männedorf, Switzerland; 4 Department of Statistics and Quantitative Methods, Unit of Biostatistics, Epidemiology and Public Health, University of Milano-Bicocca, U7, stanza 2064, 20126, Milan, Italy

**Keywords:** Patient reported outcomes, Total knee arthroplasty, Validation, Translation, Forgotten Joint Score, Arthroplasty, Ceiling effect

## Abstract

**Background:**

With ever-increasing numbers of patients undergoing elective total knee arthroplasty (TKA) and as TKA is performed in increasingly younger patients, patient demands and expectations have also increased. With improved patient outcomes, new PROMs with heightened discriminatory power in well-performing patients are needed. The present study aimed to translate and validate the Italian version of the Forgotten Joint Score (FJS-12) as a tool for evaluating pre-operative through longitudinal post-operative outcomes in an Italian population.

**Methods:**

In this prospective study, patients with unilateral osteoarthritis, undergoing TKA surgery between May 2015 and December 2017 were recruited to participate in the study. The FJS-12 and WOMAC were collected pre-operatively and at six and 12 months post-operatively. According to the COSMIN checklist, reliability, internal consistency, validity, responsiveness, effect size, and ceiling effects and floor effects were evaluated.

**Results:**

One hundred twenty patients completed the study, 66 of which participated in the evaluation of test-retest reliability. Good test-retest reliability was found (ICC = 0.90). The FJS-12 also showed excellent internal consistency (Cronbach α = 0.81). Construct validity with the WOMAC, as a measure of the Pearson correlation coefficient, was moderate (r = 0.45 pre-operatively; r = 0.46 at 6 months and r = 0.42 at 12 months post-operatively). From six to 12 months, the change was slightly greater for the WOMAC than for the FJS-12 patients (effect size d = 0.94; d = 0.75, respectively). At 12-months follow-up, the ceiling effects reflecting the maximum score were 12% for the FJS-12 and 6% for the WOMAC; however, scores within 10% of the maximum score were comprised 30% of the FJS-12 scores and 59% for the WOMAC.

**Conclusion:**

The Italian FJS-12 demonstrated strong measurement properties in terms of reliability, internal consistency, and construct validity in TKA patients. Furthermore, a more detailed look at ceiling effects shows a superior discriminatory capacity when compared to the WOMAC at 12-months follow-up, particularly in better-performing patients.

**Trial registration:**

clinicaltrials.gov NCT03805490. Registered 18 January 2019 (retrospectively registered).

## Background

Worldwide, the number of total knee arthroplasties (TKAs) is steadily increasing at annual rates ranging from 5.3% in France to 17% in Portugal [1]. In Italy, annual growth in TKA procedures has been reported to be from 6.6% [2] to 12.8% [1]. Remarkably, the number of younger TKA patients has been increasing rapidly [3]; in some populations up to a 3-fold has been seen over the last twenty years [4]. Consequently, increased patient expectations and improved outcomes have been reported [5]. Therefore, instruments which seek to measure outcomes after TKA must adapt to meet the changing expectations [6, 7].

Historically, successful interventions have been measured by concrete, surgeon-centred assessments such as range of motion, joint stability, and implant survival. In recent years, however, patient-reported outcome measurements (PROMs) have become more common and provide a new tool for evaluating the everyday life of patients after TKA [8]. The ability to discriminate between patients with good or excellent outcome from patients with poorer outcomes can be determined by implementing one of several validated scores, such as the Western Ontario and McMaster Universities Osteoarthritis Index (WOMAC) [9] and the Knee Society Score [10]; however, a considerable ceiling effect has been detected among these scores [11, 12], revealing a weakness in the capacity to differentiate between good and excellent scores. As joint arthroplasty has evolved, outcomes have continued to improve, and as a consequence patient demands and expectations have also increased. With improved patient outcomes, new PROMs with heightened discriminatory power in well-performing patients are needed [7]. In 2012, Behrend et al. introduced the FJS-12 as a means of assessing the patient’s degree of awareness of their prosthetic joint; it has since been proven to have a lower ceiling effect than other orthopaedic scores [13].

The objective of the present study is to cross-culturally adapt the FJS-12 for use in the Italian population and to test the validity, reliability, and floor and ceiling effects in a group of consecutive patients, assessed from pre-operative presentation through 1-year follow up. To our knowledge, no prior study validating an Italian version of the FJS-12 has been published.

## Methods

### Validation study

A prospective, observational study was performed to evaluate the reliability, validity and responsiveness of the Italian version of the FJS-12. First, the original FJS-12 [13] was translated using the translation and back-translation method [14]. Subsequently, the translated Italian version of the FJS-12 was validated in patients who received a primary TKA. Our inclusion criteria were the presence of unilateral knee osteoarthritis (Kellgren Lawerence scale of III-IV) requiring a TKA, no previous surgery of the lower limb, and fluency in Italian reading and comprehension. Exclusion criteria deemed ineligible any patient with a history of a previous joint prosthesis of the lower limb, previous operation of the affected knee, previous major cardiac events, current use of a walking aid, or lack of the informed consent. Based on the health status questionnaire guidelines which recommend having at least 10 patients per question [15], the sample size estimation.

For the evaluation of test-retest reliability, only the first 66 consecutive patients were involved. These patients completed the preoperative FJS-12 questionnaire twice, 2 weeks apart, prior to surgery. Our study protocol adhered strictly to the Declaration of Helsinki (EN ISO 14155:1 e EN ISO 14155:2) and to the guidelines of Good Clinical Practice and was approved by our local ethics committee in May 2015.

### Assessment instruments

#### The Forgotten Joint Score

The FJS-12 was first described in the literature by Behrend et al. in 2012 [13]. It is a PROM for patients who have undergone a TKA; it introduces the concept of a “forgotten” joint as the ideal objective to pursue in prosthetic surgery. Although other scores have been validated and shown to be reliable, they do not have adequate responsiveness and they show a high ceiling/floor effect. The FJS-12 has shown optimal discriminatory capacity and a reduced ceiling effect compared to other scores [13]. As a result of its effectiveness, the FJS-12 has since been translated in numerous languages such as but not limited to French [16], German [17], Dutch [18], and Chinese [19].

The original FJS-12 is composed of 12 items, measuring the patient’s ability to forget the presence of an artificial joint in their daily life. For each item, there is a five-points Likert scale response. The raw results are converted to a 0–100 points scale. Highest score corresponds to good outcome with the patient not aware about the presence of the prosthesis. In the case of more than 4 answers are missing, the score should not be used (Appendix) [13].

### WOMAC

The WOMAC is a clinical orthopaedic score that was first reported in 1986. The questionnaire is comprised of 24 total questions, 5 questions on pain, 2 on stiffness, and 17 on function. For each item there is a five-points Likert scale response. Total final WOMAC scores can range from 0, the lowest functional status level, to 100, the best functional status level [9].

The WOMAC score has been translated in 65 different languages, and at present is one of the most prevalently used scores in clinical orthopaedic research. In a recent review, carried out by analysing 76 articles from 22 different countries, the WOMAC once again demonstrated excellent validity and internal consistency (> 0.90) according to the consensus-based standards for the selection of health measurement instruments (COSMIN) criteria [20]. For this reason, the WOMAC was chosen as the comparison questionnaire in the present study.

### Translation and adaptation of the Forgotten Joint Score

In the present study, the official Italian translation of the FJS-12 was not used as it was not available at the time our study began. Nevertheless, our translation of the FJS-12 questionnaire from English to Italian was executed according to an internationally accepted method.

The initial translation was performed independently by two native Italian speaking orthopaedic surgeons, who were informed about the score, together with a professional translator, native Italian speaker, who was uninformed about the score. The first version was obtained by a consensus among the three translators. The backwards translation into English was performed independently by two native English speakers with a medical background. The English version hence obtained was deemed valid in a consensus meeting between all of the involved translators. The Italian version was then tested on a sample of 20 subjects with knee osteoarthritis to evaluate the comprehensibility and simplicity of the writing.

Subsequently, the validation of the translated FJS-12 score was conducted. In the validation phase, 217 patients who were to undergo a TKA were enrolled. After signing an informed consent, the patients completed the FJS-12 and WOMAC scores preoperatively, at 6 months and at 12 months post-operatively during the routine follow-up visits. The questionnaires were never filled out in the presence of the principal or sub-investigators. Patients who for geographic reasons could not complete the questionnaires at our clinic during the follow-up visits, completed the questionnaires by mail.

### Reliability

Reliability is defined as the degree to which the measurement is free from measurement error [21]. In order to assess the reliability, internal consistency, test-retest reliability, and measurement error were calculated.

### Internal consistency

Internal consistency is determined by the degree of inter-relatedness among questionnaire items [21]. A Cronbach’s α of greater than 0.7 was considered sufficient [15].

### Test-retest reliability

Test-retest reliability refers to the extent to which results from the same patient, with a static health status, remain unchanged over time [21]. According to the COSMIN manual recommendation, the retest was performed 2 weeks after the first test was administered to avoid relevant changes in health condition. Intraclass correlation coefficients (ICC) were calculated for all patients to ascertain any change of the knee condition since the first evaluation. Sufficient test-retest reliability was assumed for an ICC greater than 0.7 [15].

### Measurement error

Measurement error is the systematic and random error of a patient’s score that is not attributed to true changes in the construct to be measured [21]. The standard error of measurement (SEM) was calculated using the formula SD*√1 – ICC (SD = standard deviation) [15]. The smallest detectable change (SDC) reflects the smallest individual change in a score that can be interpreted as a real change. It was calculated with the following formula: SEM × 1.96 × √2 [15].

### Validity

The validity of the FJS-12 was measured via construct validity, which is the degree to which the scores of a questionnaire are consistent with hypotheses that the questionnaire validly measures the construct to be measured [21]. For a Pearson correlation coefficient 0.3 < r < 0.7 moderate correlation was assumed. For an r < 0.3 the correlation was considered poor. To facilitate this calculation, the questionnaire scores were divided into 10 classes before realizing the evaluation.

### Responsiveness

The capacity of a questionnaire to detect a change over time in the construct which is to be measured is referred to as the responsiveness [21]. It was calculated by measuring the change between pre-operative presentation and six-month follow-up, and between the six-months and 12-month follow-ups. This change is reported as effect size (ES) for the mean change in terms of Cohen’s d.

Furthermore, the SDC was used as a secondary measure of responsiveness. When the SDC is smaller than the minimal important change (MIC), a positive rating for responsiveness is assumed [15]. The MIC calculation was simply half of the SD [22].

### Statistical analysis

Statistical analysis was executed using the software SPSS (IMB SPSS Statistics 21, SPSS INC, Chicago, Illinois). Descriptive data are given as mean ± SD, unless otherwise stated. Ceiling effect was examined by considering the percentage of patients that achieved the maximum score (100) as well as when patients reached a score within 5 and 10% of the maximum achievable score for the FJS-12 and the WOMAC [23]. To be comparable with previous studies, if less than 15% of patients achieved the maximum score, ceiling effects were considered acceptable [15, 24].

## Results

Between May 2015 and December 2017, 217 patients were enrolled in our study. Ninety-seven patients were excluded for missing one of the two follow-up time points. Of the remaining 120 patients that could be included in our study, 39 (32.5%) were men and 81 (67.5%) were women. The mean age was 70.0 ± 8.4 years (range 44–86). The mean interval for the 6-month post-operative follow-up was 6.5 ± 0.8 months (range 5–8 months); and the mean interval for the final follow-up at 1 year was 12.5 ± 1.2 months (range 10–15). The FJS-12 and WOMAC pre-operative, 6-month follow-up, and 12-month follow-up outcomes are summarized in Table 1. To evaluate the test-retest reliability, the first 66 patients were included and the time span between the first and second pre-operative questionnaire was 14 days ±2 days.

**Table 1:** Mean scores for FJS-12 and WOMAC at all time points

	Pre-op	6 m follow-up	12 m follow-up
FJS-12	24.5 ± 16.6	60.5 ± 25.0	73.1 ± 23.4
WOMAC	41.1 ± 16.8	74.0 ± 18.4	85.6 ± 15.7
1-way ANOVA	*p* < 0.001	p < 0.001	p < 0.001

14 total complications were recorded. There were 12 cases (10%) of anaemia, 1 case (0.8%) of acute pulmonary oedema and atrial fibrillation, and 1 case (0.8%) of hypoglycaemia.

A Cronbach’s α of 0.81 (95% CI: 0.76–0.86, *p* < 0.001) indicated excellent internal consistency for the FJS-12. As this value is greater than 0.7, sufficient internal consistency was assumed. The test-retest reliability was also good for all patients, with an ICC of 0.90 (95% CI: 0.86–0.92, *p* < 0.001) The SEM for the total FJS-12 was 5.25; therefore, the SDC, reflecting the smallest individual change in a score that can be considered as real change, was 14.55.

The Pearson correlation coefficient between the FJS-12 and WOMAC was 0.45 (*p* < 0.001) at pre-op, r = 0.46 (*p* < 0.001) at 6 months, and r = 0.42 (*p* < 0.001) 12 m. Therefore, construct validity showed a moderate correlation between the two functional scores at all time points.

**Fig. 1:**
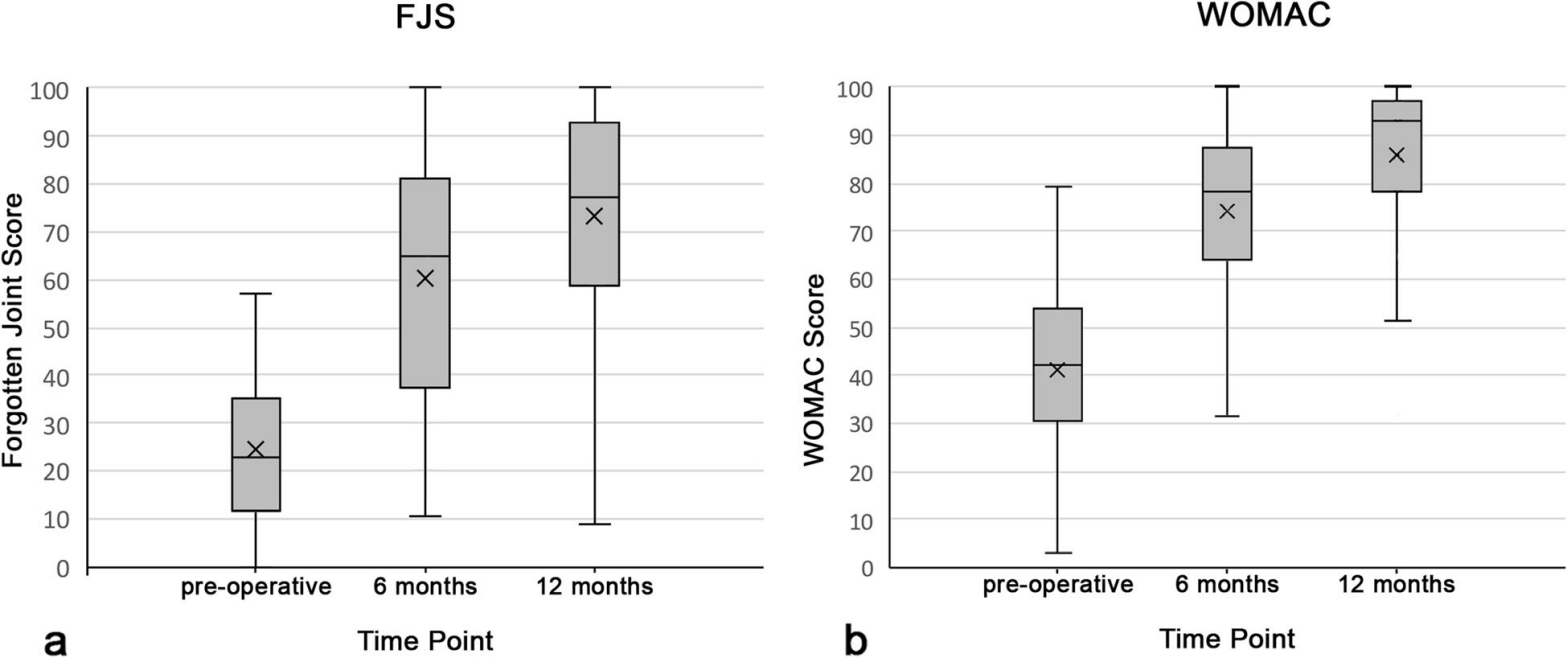
Boxplot of the Forgotten Joint Score (**a**) and WOMAC (**b**) pre-operatively, and at 6 and 12 months post-operatively. Boxplots of the Forgotten Joint Score (**a**) and the WOMAC score (**b**). The “X” indicates the mean score; the line inside the box indicates the median score

To measure the performance of the FJS-12 over time, the data was analysed at all time-intervals after surgery (Fig. 1). The results are detailed in Table 1. Preoperatively, the mean FJS-12 score was 24.4, and at 6 months the mean was 60.5 (Table 1), resulting, as expected, in a very large ES for the time span from pre-operative to six-month follow-up (Cohen’s d = 1.84). The WOMAC also demonstrated a very large ES (Cohen’s d = 1.29), with mean pre-op scores of 41.1 and mean 6-month follow-up scores of 74.0. For the interval between the six month and one-year follow-up, a medium effect size (d = 0.75) was found for FJS-12 and a high effect size (d = 0.94) for WOMAC. Lastly, as the pre-operative MIC was 8.3, while the pre-operative SDC was 14.55, according to Terwee et al. [15], no positive rating for responsiveness could be given as SDC was greater than MIC. The MICs for both the FJS-12 and WOMAC at all three time points can be found in Table 2.

**Table 2:** Minimal important change (MIC) for FJS-12 and WOMAC at all time points

	Pre-op	6 m follow-up	12 m follow-up
FJS-12	8.3	12.5	11.7
WOMAC	8.4	9.2	7.8

**Fig. 2:**
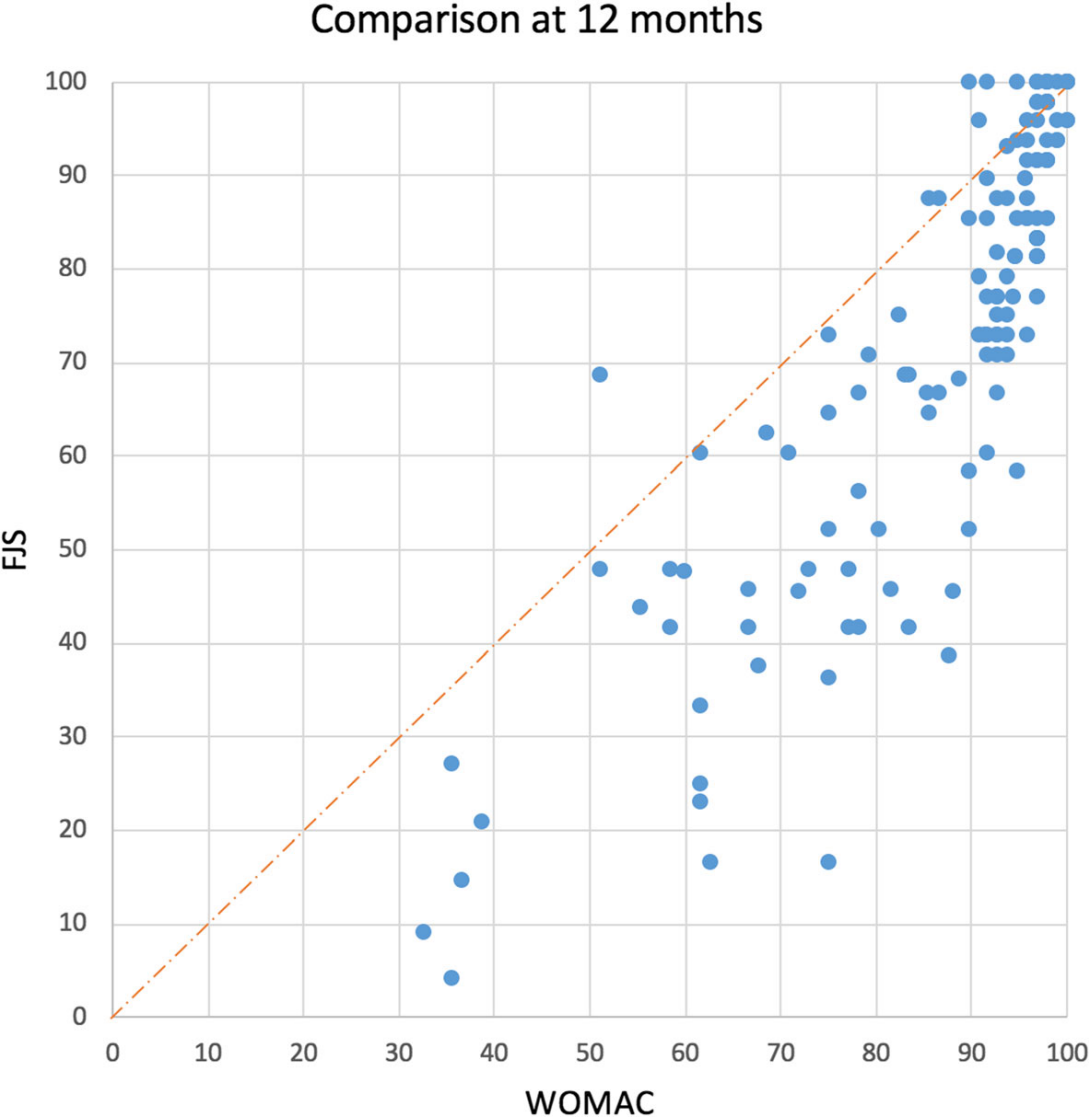
Comparison of the Forgotten Joint Score and the WOMAC at 12-months follow-up. Comparison of the12-month follow up scores for the FJS-12 and WOMAC. Each point represents the scores of one single patient; scores were plotted consecutively by date of intervention

When the maximum score of 100 was used as the cut-off for measuring the ceiling effect at the 12-month follow-up, the ceiling effect was less than 15%, and therefore acceptable, for both the FJS-12 (12%) and WOMAC (6%). However, when plotted, a visible “ceiling” can be seen in both scores (Fig. 2), although the FJS-12 succeeds in distributing the scores more evenly than the WOMAC. Plotting a bisector, reveals that most subjects have a lower FJS-12 than WOMAC score, confirming the hypothesis of FJS-12 having a lower ceiling effect. Table 3 shows the ceiling effects at three different cut-off scores. At 12 months post-operatively, more than a third of WOMAC scores were above 95, compared to 19% of the FJS-12 scores. This disparity greatens further when the cut-off is lowered to scores greater than 90, which comprised more than half (59%) of the WOMAC scores and only 30% of the FJS-12 scores.

**Table 3:** Ceiling effects at 12-months follow-up for the FJS-12 and WOMAC

Maximum score (100)		score of > 95%		score of > 90%	
FJS-12	WOMAC	FJS-12	WOMAC	FJS-12	WOMAC
12%	6%	19%	34%	30%	59%

Based on the pre-op scores, both the FJS-12 (5%) and WOMAC (0%) were in the acceptable range (of < 15%) for floor effects. Furthermore, at 12 months follow-up, 0 patients recorded the minimum score.

At all three time points, question 12, was left unanswered by a large number of patients (21.7% at pre-op, 14.2% at 6 months post-op, and 7.5% at 12 months post-op). Question 7 was left unanswered by 2.5% at pre-op and 6 months post-op and 0.8% at 12 months post-op. Finally, question 4 was left unanswered by 3.3% of patients only at pre-op.

## Discussion

In order for patients to “forget” their artificial knee, in addition to being free of pain, it must offer an acceptable range of movement and provide stability during daily activities. The original FJS-12 is a PROM that comprises all three of these parameters in the assessment of the post-operative outcome.

This is the first study to validate the Italian version of the FJS-12 using the COSMIN checklist [21]. Sufficient validity and reliability in the Italian version of the FJS-12 were confirmed. Moderate correlation was found between the FJS-12 and the WOMAC at all time points. According to Terwee and colleagues [15], the FJS-12 did not achieve a positive rating for responsiveness as the MIC (8.3) was higher than the SDC (17.7). However, for FJS-12, effect size was very large between pre-op and six months (1.84), and from 6 months to 12 months there was a medium effect size (d = 0.75). Furthermore, Although the FJS-12 was originally intended to measure post-operative outcomes, our study shows that it can also be used across the patient’s entire surgical pathway, evaluating pre-operative and longitudinal post-operative outcomes.

The mean age of 70 years for patients in our study is comparable to the average age in other knee arthroplasty studies [13, 25]. The substantial number of patients (21.7% at pre-op and 7.5% at 12-month follow-up) who left question 12, regarding sports, unanswered has been noted in other studies of FJS-12, such as in the French study where 40% of patients left question 12 unanswered. In future versions of the questionnaire, it would be wise to consider rephrasing this question to improve the applicability for Italians and increase the likelihood of subjects to complete this item.

The original FJS-12 publication found a Cronbach’s α of 0.95, and a positive rating for internal consistency was given [13]. Unlike the original FJS-12 study, in the present study, test-retest reliability was evaluated pre-operatively, and excellent internal consistency was demonstrated via a Cronbach’s α of 0.81. Likewise, two other validation studies, one Danish [26] and one German [17], both found a high level of internal consistency (Cronbach’s α = 0.96 and Cronbach’s α = 0.95, respectively). Several other studies have investigated test-retest reliability via calculation of the ICC, all finding high internal consistency.^17,1819,26^ In the present study, good test-retest reliability was confirmed with an ICC of 0.90 [15]; this value was comparable to the German study’s ICC of 0.80 [17], the Dutch study’s ICC of 0.94 [18], and the Chinese study’s ICC of 0.97 [19], and the Danish study’s ICC of 0.91 [26].

Similar to the original FJS-12 [17], it was assumed that the WOMAC, which is widely used in Italy, would be a good tool with which to compare the FJS-12. However**,** unlike the high correlation between FJS-12 and WOMAC seen in previous studies previous studies [13, 27], we found only a moderate correlation between the two functional scores. The differences in the phrasing of the items among the two questionnaires makes it unsurprising that only a modest correlation would be found.

Between pre-operative presentation and 6 months post-operatively, the FJS-12 demonstrated a very large effect size (Cohen’s d = 1.84) that was slightly larger than the effect size found by the WOMAC (Cohen’s d = 1.29). However, for the interval between 6 months and 12 months post-operatively only a medium effect size was found (Cohen’s d = 0.75), and this change was slightly less than the change detected via the WOMAC (Cohen’s d = 0.94). Our results differ from Hamilton et al.’s 2017 study in which they found the FJS-12 to be more responsive in the latter period of follow-up when compared to another Orthopaedic score; however, their effect size (Cohen’s d = 0.12) was much smaller than in our study.

According to Terwee et al. [15], no positive rating for responsiveness could be given for the Italian version of the FJS-12 as our MIC (8.3) was less than our SDC (17.7). Our result was comparable with Bauman et al.’s findings (MIC = 10.9; SDC = 13.1), while Ingelsrud et al. [28] found a higher value for MIC [14].

The WOMAC is one of the most commonly used Orthopaedic scores and it has been pivotal in the measurement of arthroplasty outcomes. Similar to several other orthopaedic scores, the WOMAC is valid and reliable, however, these scores may suffer from high ceiling effects [29, 30] and may therefore overlook relevant changes in post-operative patient function over time, especially among patients with better outcomes. Therefore, the development of more sensitive tools for assessing arthroplasty outcomes is of growing interest [31, 32]. Moreover, as the number of younger patients with increased expectations is growing, instruments with which to measure outcomes must adapt to better distinguish between good and excellent results. The original FJS-12 paper [13] as well as several other validations studies [17, 18, 27] found no relevant ceiling effects. In our study of 120 patients, acceptably low ceiling and floor effects were found. Our ceiling effects at 12-months follow-up (12%) were similar to those found in the original FJS-12 paper (9.2%). The floor effect was 0% at 12 months, which was also similar to Behrend’s 3.3% [13].

Based solely on the maximum score (100), the FJS-12 showed a higher ceiling effect than WOMAC. However, the plotted scores reveal a more complex view of the ceiling effect. Nearly twice as many WOMAC scores were contained within the ≥90% of the maximum score; hence, FJS-12 was actually more effective at stratifying the scores in the upper ranges. We would recommend that future papers seeking to measure the ceiling and floor effects of orthopaedic questionnaires examine the effects graphically as a ceiling or a floor effect may be present even when the maximum score is not reached.

In one recent publication, the FJS-12 was used in a retrospective cohort study of Italian patients who had undergone unicompartmental knee arthroplasty (UKA) [33]. Although our TKA patients cannot be directly compared to the UKA patients in Zambianchi et al.’s study, the present study’s pre-operative and post-operative results were similar to their results; however, their pre-operative scores were on average slightly lower and their post-operative scores were on average slightly greater than our scores [34]. Such differences in baseline scores may also reflect different indications for surgery or differences in health status in the general populations.

In the two other studies that included pre-operative data [35, 36], our data (24.5 ± 16.6) was more similar to the data of the French population (24 ± 16) than the British population (11.5 ± 11.6). Likewise, considering the one-year follow-up data, our results (73.1 ± 23.4) were very similar to the French study’s results (70 ± 27) and quite different from the British results (45.7 ± 31.2). Cross-cultural variability may have affected the disparities among the results [37].

Our prospective study included 120 TKA patients. The principal limitation in our study was our high drop-out rate. Our study is strengthened by the decision to include only TKA patients with primary knee osteoarthritis to create a more homogenous sample. Furthermore, none of the patient’s knees had been previously operated for any other pathology. Finally, this is one of the first studies to evaluate the FJS-12 from pre-operative presentation through post-operative follow-ups [35, 36]. Although the FJS-12 was developed as a post-operative measurement tool, if future studies also collected pre-operative data, it would provide reference point to which post-operative outcomes could be compared, providing a more complete understanding of the success of joint arthroplasty. As low floor effects were found at pre-operative presentation, the FJS-12 may be suitable for use pre-operatively as well as post-operatively.

## Conclusion

In summary, the FJS-12 demonstrated good test-retest reliability and was moderately correlated with the WOMAC. For scores within 5 and 10% of the maximum score, the FJS-12 had a lower ceiling effect than the WOMAC. Hence, this Italian version of the FJS-12 questionnaire seems to be an acceptable representation of the original questionnaire, and we would recommend its use in research concerning the outcomes of TKA as well as other joint arthroplasties in Italy. With increasing numbers of TKAs and increasing patient expectations, the capacity of the FJS-12 to distinguish between patients with good or excellent results after TKA could be advantageous.

## Data Availability

The datasets used and/or analysed during the current study are available from the corresponding author on reasonable request.

## Appendix

### Forgotten Joint Score

**Table 4:** Questo questionario serve all’equipe dei suoi medici curanti a valutare quanto la sua articolazione o protesi si “*fa sentire*” durante le attività quotidiane. La preghiamo di compilarlo con la massima cura e sincerità possibile

	MAI	Quasi mai	Raramente	Qualche volta	Sempre
1) senti la protesi a letto durante la notte					
2) senti la protesi quando stai seduto su una sedia per più di un’ora?					
3) senti la protesi quando cammini per più di 15 minuti?					
4)senti la protesi quando fai la doccia/bagno?					
5) senti la protesi quando viaggi in macchina?					
6)senti la protesi quando sali le scale?					
7) senti la protesi quando cammini su un terreno sconnesso?					
8) senti la protesi quando ti alzi da seduto?					
9) senti la protesi quando stai in piedi per un lungo periodo di tempo?					
10) senti la protesi quando fai i lavori di casa?					
11) senti la protesi quando fai una camminata?					
12) senti la protesi quando fai sport o hobby?					

## Abbreviations

COSMIN: COnsensus-based Standards for the selection of heal Measurement Instruments
ES: Effect size
FJS-12: Forgotten joint score
FJS-12: Italian forgotten joint score
ICC: Intraclass correlation coefficients
MIC: Minimal important change
PROM: Patient-reported outcome measurement
SD: Standard deviation
SDC: Smallest detectable change
SEM: Standard error of measurement
TKA: Total knee arthroplasty
WOMAC: Western Ontario and McMaster Universities Osteoarthritis Index
